# Numerical Simulation of Abandoned Gob Methane Drainage through Surface Vertical Wells

**DOI:** 10.1371/journal.pone.0125963

**Published:** 2015-05-08

**Authors:** Wei Qin, Jialin Xu, Guozhong Hu

**Affiliations:** State Key Laboratory of Coal Resources and Mine Safety, School of Mines, China University of Mining and Technology, Xuzhou, Jiangsu Province, China; China University of Mining and Technology, CHINA

## Abstract

The influence of the ventilation system on the abandoned gob weakens, so the gas seepage characteristics in the abandoned gob are significantly different from those in a normal mining gob. In connection with this, this study physically simulated the movement of overlying rock strata. A spatial distribution function for gob permeability was derived. A numerical model using FLUENT for abandoned gob methane drainage through surface wells was established, and the derived spatial distribution function for gob permeability was imported into the numerical model. The control range of surface wells, flow patterns and distribution rules for static pressure in the abandoned gob under different well locations were determined using the calculated results from the numerical model.

## Introduction

Methane remaining in the gob on cessation of underground coal mining operations is defined as abandoned gob methane (AGM). AGM is a strong greenhouse gas, and its 100-year global warming potential is 21 times that of carbon dioxide [1–3]. Under the influence of changes in atmospheric pressure, AGM can escape into the atmosphere through a poorly sealed wellhead, faults or even cracks, further exacerbating the global greenhouse effect [4]. On the other hand, AGM is a source of clean energy. Therefore, the exploitation of AGM directly benefits the environment by reducing greenhouse gas emissions to the atmosphere and favors the community by providing clean energy.

The technology of AGM drainage has been drawn increasing attention recently. In the late 1990s, commercial application of AGM drainage was achieved in the United Kingdom [5]. The USA investigated the methods to evaluate the potential of AGM drainage [6]. Raven Ridge Resources, Inc. (USA), developed a software system that can be used to simulate and calculate AGM reserve [7–8]. Research on AGM drainage in China was mainly limited to AGM resource reserve evaluation, [9–12]. In conclusion, many scholars have paid much attention to reservoir strata description of AGM, but there is little concern on gas seepage characteristics in abandoned gob.

This paper investigated the gas seepage characteristics in the abandoned gob where surface well drainage was conducted. A physical modelling was performed to study the movement of overlying strata, based on the actual conditions in a working face. A spatial distribution function for the vertical displacement of the overlying strata in a gob was proposed. On this basis, a spatial distribution function for gob permeability was derived. A numerical model using FLUENT for abandoned gob methane drainage through surface wells was established, and the derived spatial distribution function for gob permeability was imported into the numerical model. The control range of surface wells, flow patterns and distribution rules for static pressure in a gob under different well locations were determined using the calculated results from the numerical model.

## Three-dimensional physical simulation of movement of the overlying strata

### 2.1 Prototype of the simulated working face

**Fig 1:**
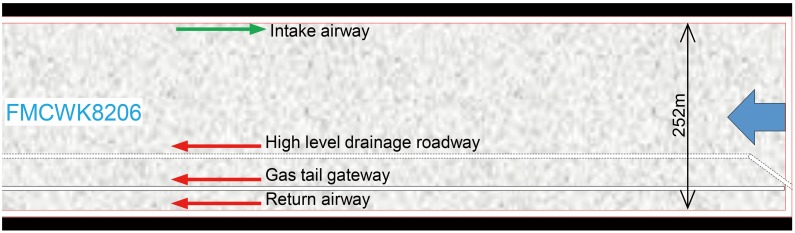
Roadway layout of FMCWK8206.

The prototype of this simulation experiment is based on the K8206 fully mechanized caving workface (FMCWK8206) in Yangquan Coal Mine No. 3, China (Fig 1). FMCWK8206 extracts the No. 15 coal seam, for which the average dip, thickness and depth are 5°, 7.08 m, and 520–572 m, respectively. The incline width of FMCWK8206 is 252 m, and the strike advancing length is 1579 m. Retreat mining is used, and the roof is managed by the full caving method.

### 2.2 Experimental device

**Fig 2:**
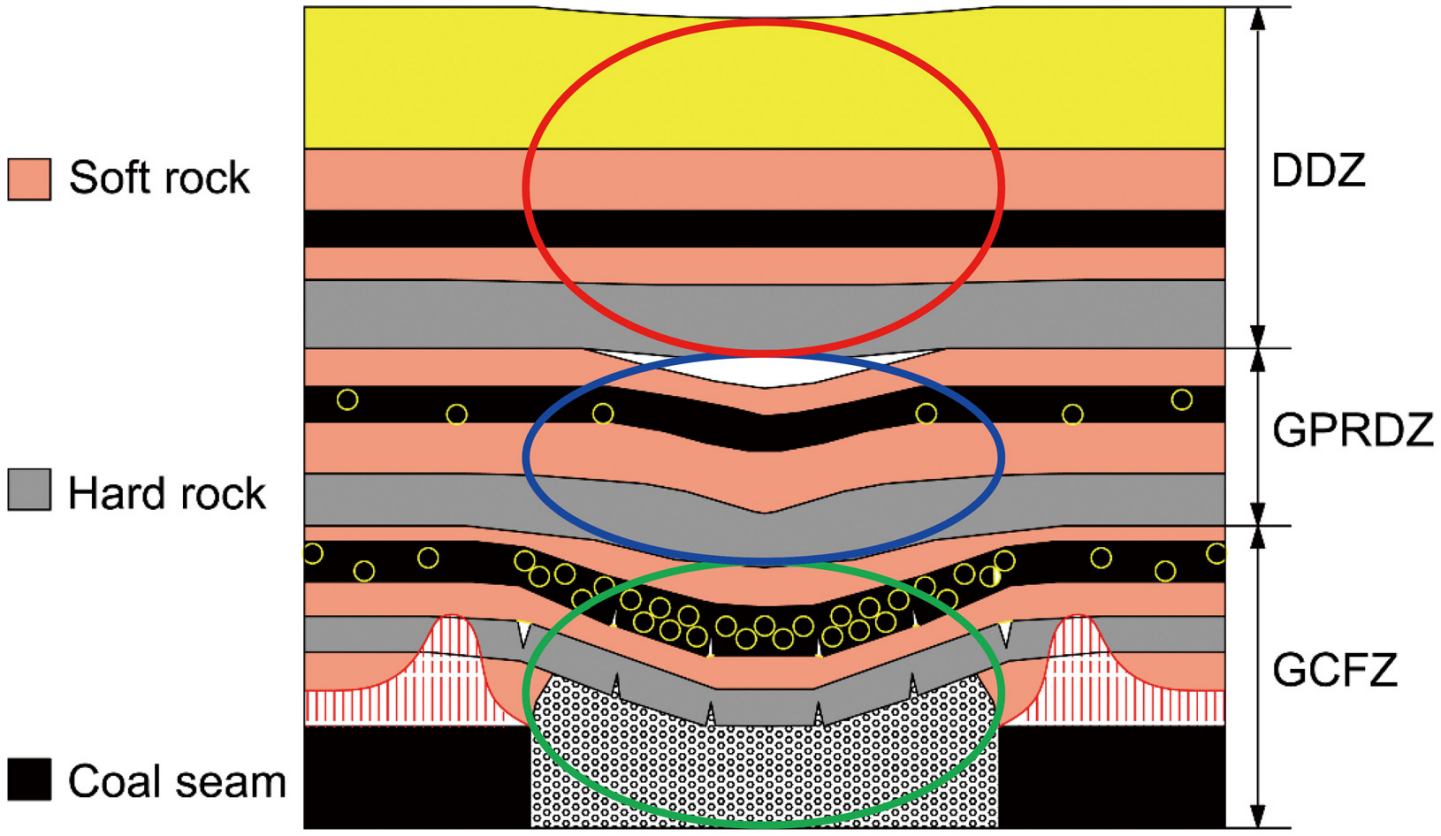
Division of the overlaying strata into three zones divided by relieved gas delivery.

The overlying strata are divided into three zones [13]: the gas conductive fracture zone (GCFZ), the gas pressure relief and desorption zone (GPRDZ), and the difficult desorption zone (DDZ) (Fig 2). There are massive connected fissures within the range of the GCFZ. Coal seams and strata can be released fully within the range of the GPRDZ. Coalbed methane (CBM) retains its original state in the DDZ. In the study of R.L. Wu [14], the height of the GCFZ of FMCWK8206 is 125 m, and the height of the GPRDZ is 173 m.

**Fig 3:**
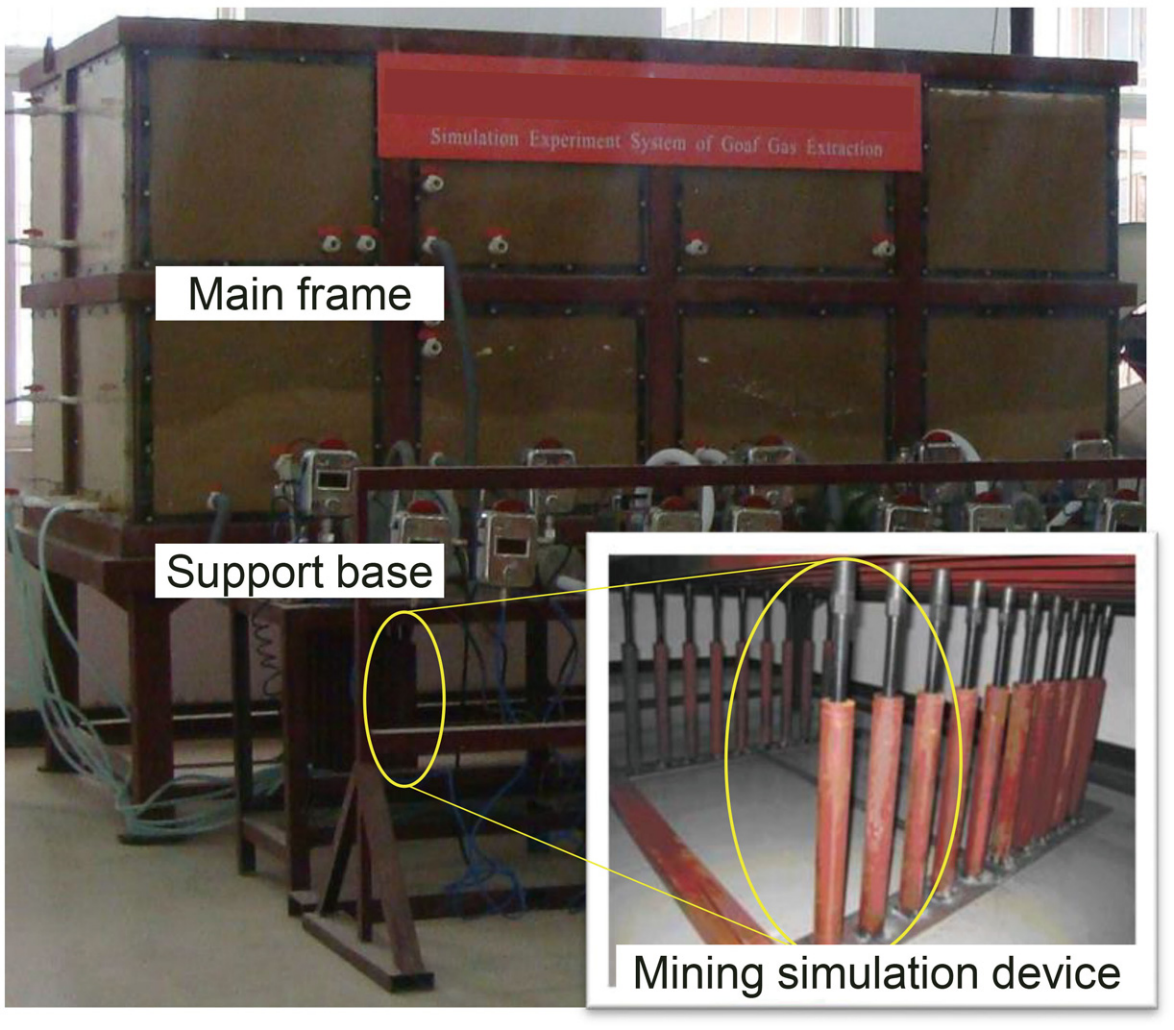
Three-dimensional simulation system for a gob.

A custom-made three-dimensional simulation system for a gob was used for this experiment. The main structure of this device, which is shown in Fig 3, includes three parts: the main frame, the supporting base and the mining simulation device. The geometric similarity ratio between this experimental model and the actual working face is 1:100. According to the similarity ratio, the length of the working face in the model is 2.52 m, and the laying height of the model is 1.25 m. The length of the working face along the advancing direction is 1.3 m, and the mining height is 0.068m. The weight of coal petrography in the GPRDZ is converted into the vertical load on the top interface of this model.

In this experimental device, lifting screws were used to simulate the mining of a coal seam. The falling of the screws caused the drop of loose bodies from the caving zone to the bottom of the main part in the model, which provided the sinking and revolving space for the upper precast block. With the advancement of the simulated working face, the upper test block was periodically loosened and rotated. In total, there were 10 rows of screws, with two screws in each row. The width of the supporting steel plate for each row of screws was 0.13 m. The complete drop for each row of screws corresponded to the advancement of the working face by 13 m.

### 2.3 Experimental scheme

#### 2.3.1 Simulated materials

**Fig 4:**
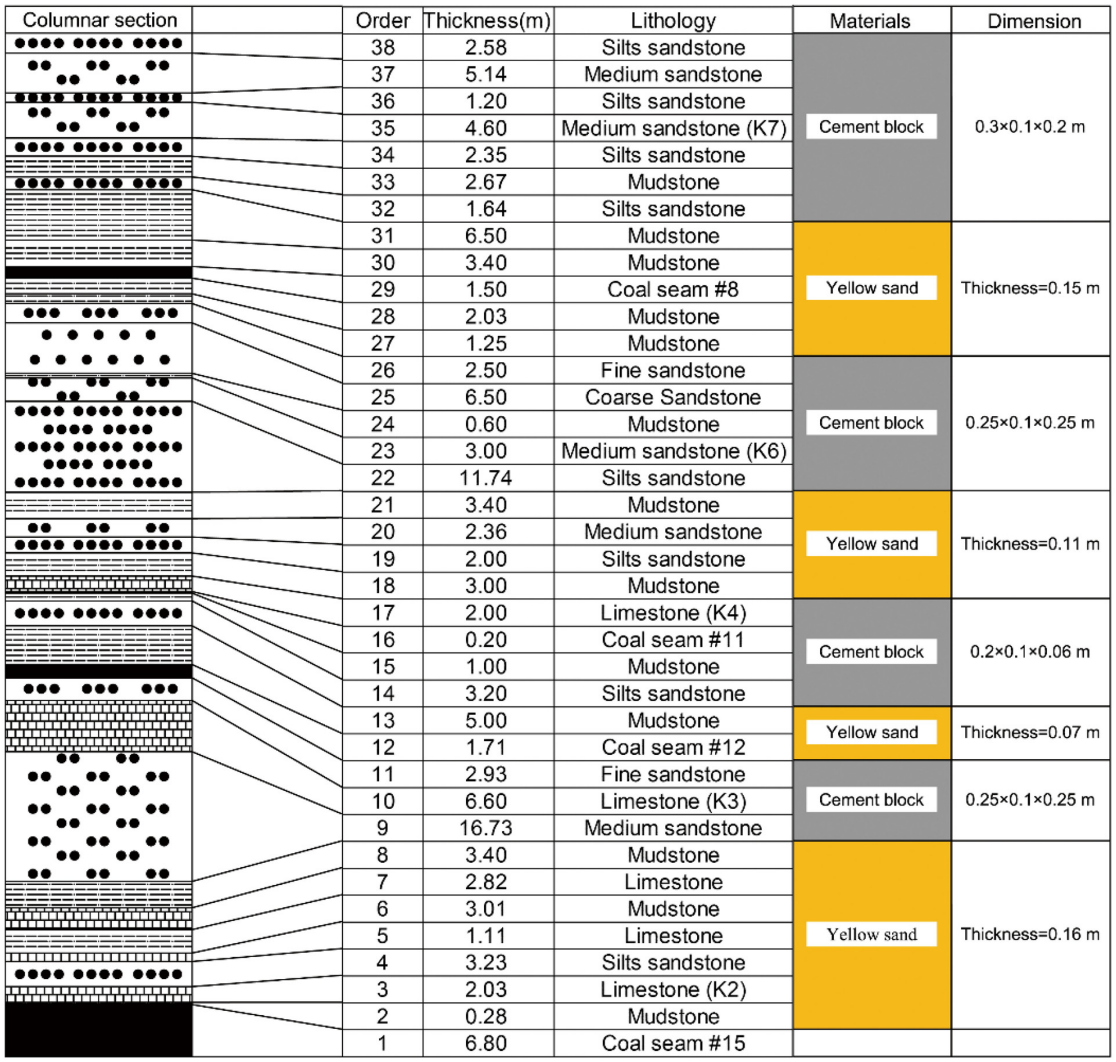
Grouping of the overlying rocks and the arrangement of the simulated materials.

Yellow sand and cement precast blocks were used to simulate the soft and hard rock stratums in the GCFZ, respectively. The size of the cement precast blocks was determined by the pitch of the fractured step of the hard rock strata. The grouping of the overlying rocks and the arrangement of the simulated materials are shown in Fig 4.

#### 2.3.2 Measuring lines

**Fig 5:**
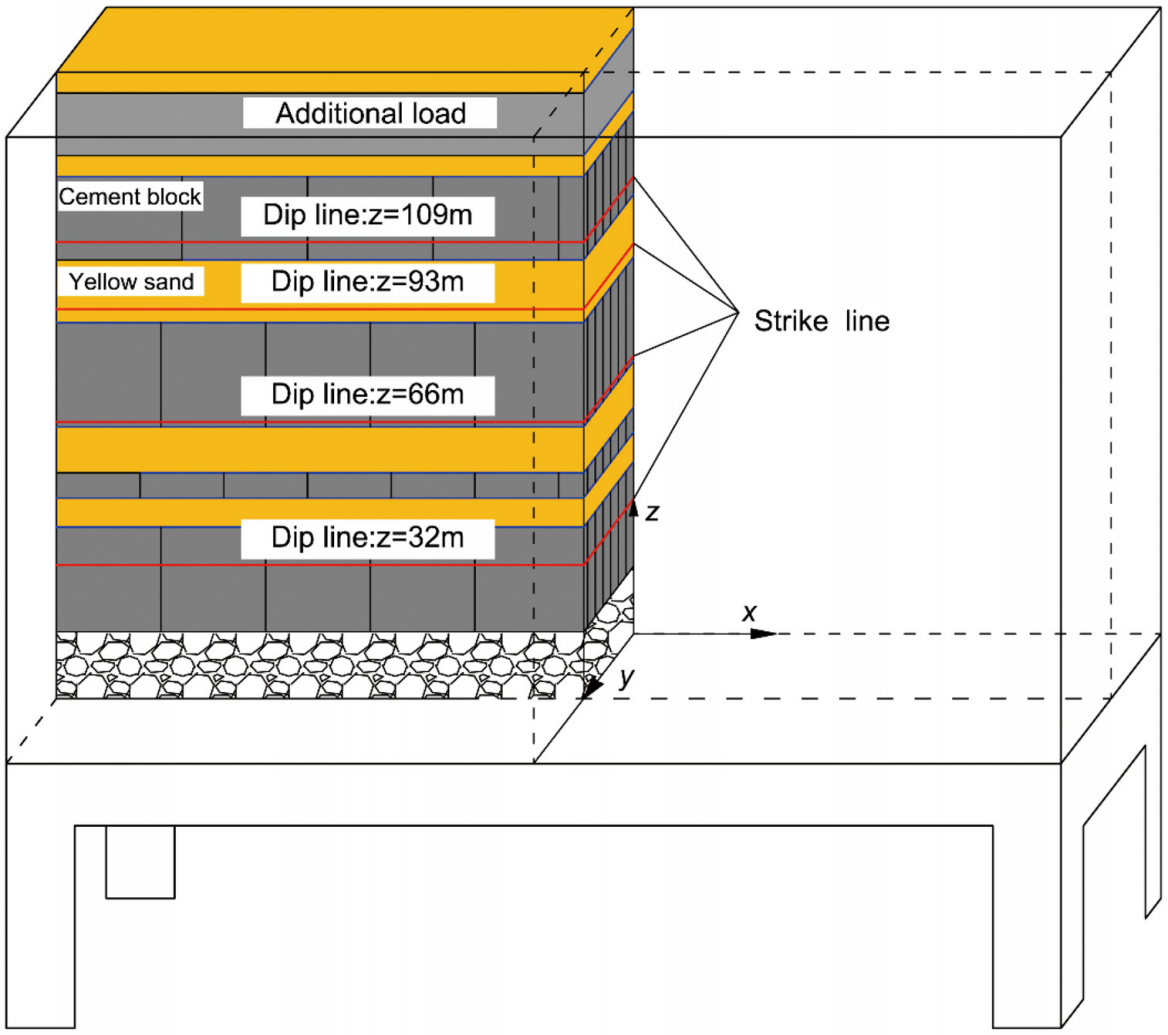
Layout of the measuring lines.

In this test, the measuring lines were wired onto the main fracture sections at *z* = 32, 66, 93 and 109 m horizons. Due to the symmetry of the gob, the measuring lines for the vertical displacements of the rock strata were unilaterally arranged. The wiring scheme for the measuring lines is shown in Fig 5.

### 2.4 Analysis of the experimental results

#### 2.4.1 Curve of rock strata movement along the strike direction

The curve of movement inside the rock stratum corresponds to the displacement curve of each point inside the rock strata in the vertical direction. The vertical displacement of the overlying rock strata in a gob is related to its spatial location by a continuous function. Along the strike direction of the working face, according to the theory of a masonry beam [15], the curvilinear equation of internal movement for rock stratum is

$$W_{y^{\prime }}=W_{0}\left[1-\exp\left(-\frac{y^{\prime }}{2l}\right)\right]$$ (1)

where *W*_*y′*_ is the vertical displacement of the rock stratum (m), *y′* is the distance to the working face (m);$l=h\sqrt{R_{\text{T}}/3q}$, where *R*_T_ is the tensile strength of the rock stratum (Pa), *h* is the thickness of the rock stratum (m) and *q* is the self-weight and load from the upper strata (Pa); and *W*_0_ is the theoretical maximum vertical displacement of the overlying rock strata (m), where a negative value for *W*_0_ indicates subsidence.

**Fig 6:**
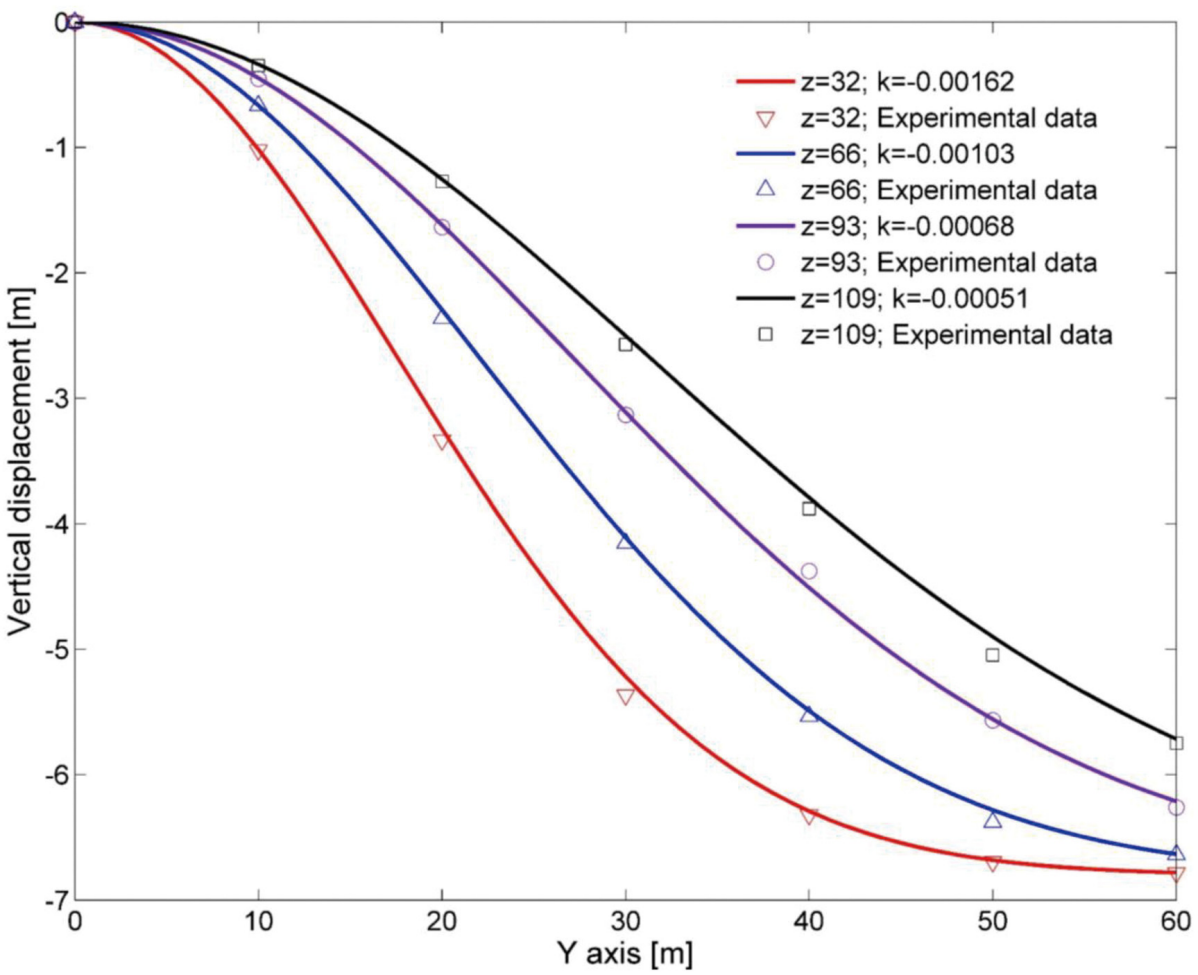
Fitting curves for vertical displacement of the overlying strata in the strike direction principal section.

The maximum vertical displacement of the overlying rock strata in the theoretical analysis was taken as *W*_0_ = −6.8m, which is the mining height of FMCWK8206. The vertical displacements of the measuring lines were fitted with corresponding locations as shown in Fig 6.

In order to improve the correlation of fitting, the fitting function for the vertical displacement of the rock stratum along the strike direction was slightly different from Eq (1):

$$W=-6.8[1-\exp(ky^{2})]$$ (2)

where *k* is the fitting coefficient and *y* is the distance to the open-off cut along the strike direction (m).

Fig 6 shows that the vertical displacements of each rock stratum near the open-off cut were smaller than in the middle portion. The smaller displacement results from the supporting action provided by the coal pillar. In general, the further the distance from the open-off cut, the larger the subsidence of each rock strata, and the middle region of the gob is mostly compacted. Along the strike direction of the working face, the area of the compacted zone is reduced from the bottom to the top.

In the principal section along the strike direction in the gob, the fitting coefficient of the vertical displacement function (*k*) is, respectively, −0.00162, −0.00103, −0.00068 and −0.00051 on the horizon of the upper rock strata when the values of *z* are 32, 66, 93 and 109 m, respectively. Consequently, the relationship between the fitting coefficient and the height of the corresponding horizon can be approximated by an exponential function as

$$k=-0.002581\exp\left(-\frac{z}{69.6}\right)$$ (3)

According to Eqs (2) and (3), the vertical displacement function of the rock stratum in the principal section along the strike direction of gob can be obtained as

$$W(y,z)=\left\{ \begin{array}{l} -6.8\left\{1-\exp\left[-0.002581\exp\left(-\frac{z}{69.6}\right)y^{2}\right]\right\}\\ \left(0\leq y\leq 65\right)\\ -6.8\left\{1-\exp\left[-0.002581\exp\left(-\frac{z}{69.6}{\left(130-y\right)}^{2}\right)\right]\right\}\\ \left(65\leq y\leq 130\right) \end{array}\right.$$ (4)

#### 2.4.2 Curve of rock strata movement along the dip direction

The spatial distribution function for the vertical displacement of the overlying rock strata in the gob is a function not only of *z* and *y* but also of *x*. Thus, the function can be modified to express the vertical displacements of the entire gob:

$$W^{\prime }(x,y,z)=\left\{ \begin{array}{l} -6.8\left\{1-\exp\left[-0.002581\exp\left(-\frac{z}{69.6}\right)y^{2}g(x)\right]\right\}\\ \left(0\leq y\leq 65\right)\\ -6.8\left\{1-\exp\left[-0.002581\exp\left(-\frac{z}{69.6}\right){\left(130-y\right)}^{2}g(x)\right]\right\}\\ \left(60\leq y\leq 130\right) \end{array}\right.$$ (5)

Eq (5) needs to satisfy the following conditions:

1. when *x* = 0, g(*x*) = 1, and then *W′(x*,*y*,*z)* = *W(y*,*z)*, namely the vertical displacement of the rock strata in the principal section along the strike direction;
2. when *x* = ±126, g(*x*) = 0, and then *W′* = 0, namely the vertical displacement of the rock strata at the mining boundary is 0 m.

Based on the above assumptions, $g(x)=\left(1-{\left(\frac{x}{126}\right)}^{2}\right)$ meets the above conditions. After inserting g(*x*) into Eq (5), the expression for the vertical displacements of the rock strata in the whole gob can be obtained as

$$W^{\prime }(x,y,z)=\left\{ \begin{array}{l} -6.8\left\{1-\exp\left[-0.002581\exp\left(-\frac{z}{69.6}\right)y^{2}\left(1-{\left(\frac{x}{126}\right)}^{2}\right)\right]\right\}\\ \left(0\leq y\leq 65\right)\\ -6.8\left\{1-\exp\left[-0.002581\exp\left(-\frac{z}{69.6}\right){\left(130-y\right)}^{2}\left(1-{\left(\frac{x}{126}\right)}^{2}\right)\right]\right\}\\ \left(65\leq y\leq 130\right) \end{array}\right.$$ (6)

**Fig 7:**
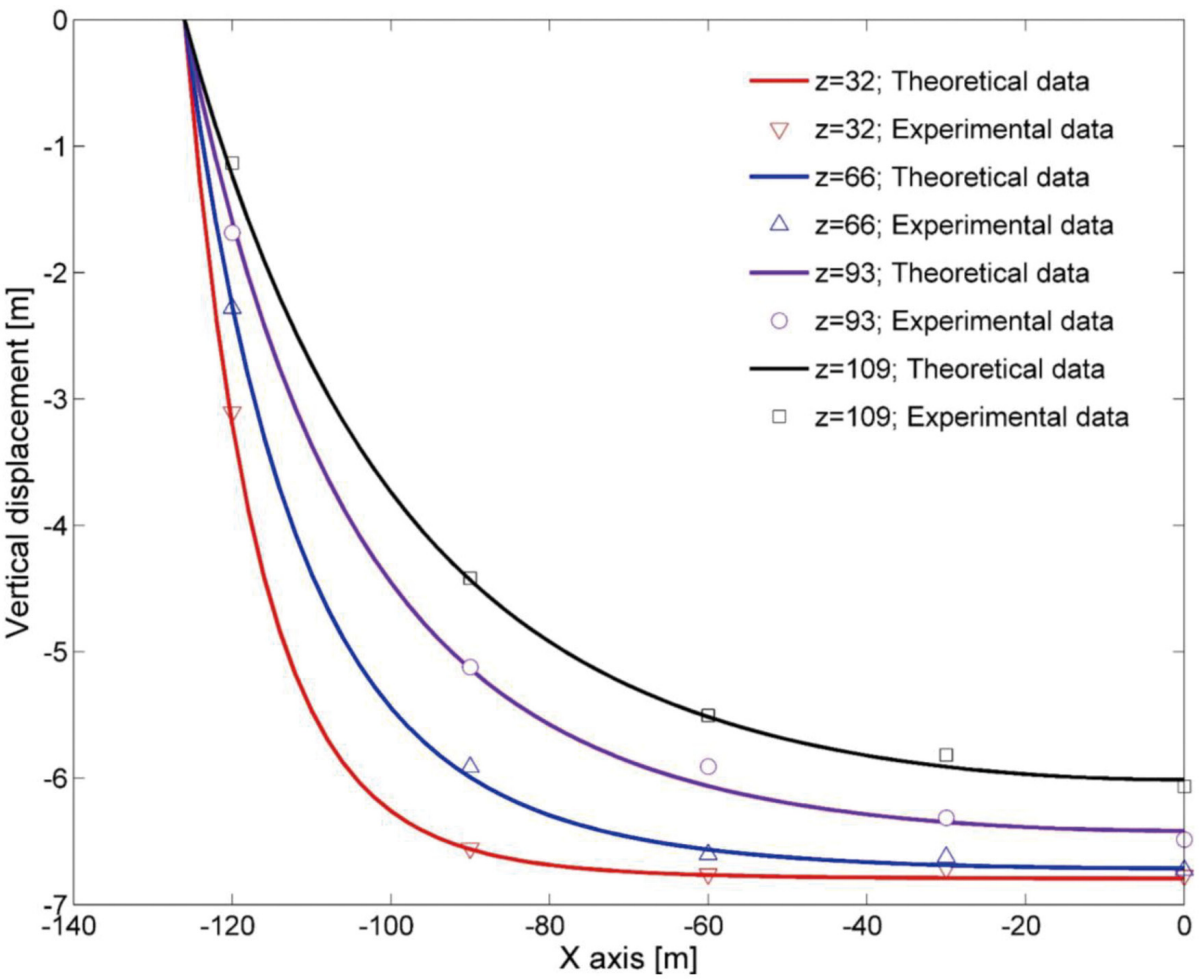
Comparisons between the results from the theoretical calculation of vertical displacement and the experimental results.

In order to investigate the accuracy of Eq (6), the calculated vertical displacement of the rock strata in the principal section along the dip direction was compared and verified with the experimental data. The comparison between the theoretical values and the experimental results is shown in Fig 7, which shows that the theoretical values are in good agreement with the experimental results. Eq (6) therefore accurately reflects the distribution of the vertical displacement for the rock strata in the entire gob. This expression was then used as the basis for the determination of the spatial distribution function of porosity and permeability within the gob.

## Spatial distribution of gob permeability

Gob permeability is an important factor that influences gas seepage in a gob. The basis for understanding the characteristics of gas seepage in a gob is dependent on accurately describing the spatial distribution of gob permeability. Despite the importance of this reservoir property, its prediction is difficult, and has been conducted in only a few studies [16–19]. This may be due to challenges and unknowns related to the gob environment. These challenges are made even more difficult due to the inaccessibility of the gob environment for conducting direct measurements of permeability using conventional tests [20]. In this paper a spatial distribution function for the vertical displacement of the overlying strata in gob was proposed. On this basis, a spatial distribution function for the gob permeability is derived.

### 3.1 Spatial distribution function of gob permeability

**Fig 8:**
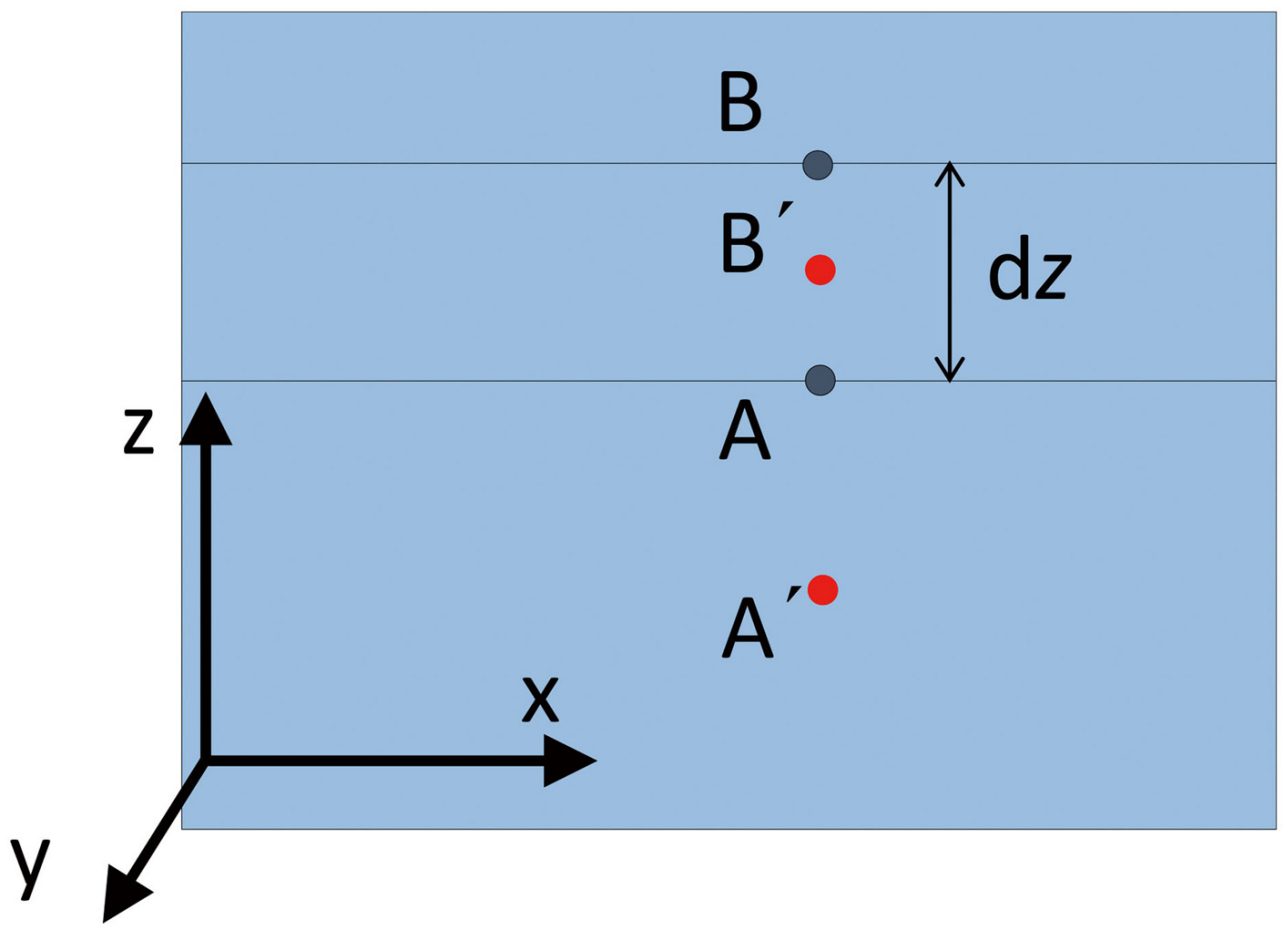
Two points in the vertical section of the gob.

Before the coal seam was mined, two points A(*x*_0_, *y*_0_, *z*_0_) and B(*x*_0_, *y*_0_, *z*_0_ + d*z*) were taken in the vertical direction of the overlying coal stratum. The link line between the two points is parallel to the *z* axis and perpendicular to the *x*–*y* plane, as shown in Fig 8.

After the coal seam was mined, points A and B sunk to A′ and B′, respectively. According to Eq (6), the vertical displacements of A and B are *W*′(A) and *W*′(B), respectively. As the subsidence of the two points is not synchronised, the space formed between points A and B, after the coal seam is mined, is *H*_space_ = *W′*(A)−*W*′(B), while the porosity between points A and B is as follows:

$$\phi =\frac{H_{\text{space}}}{\text{d}z}=\left|\frac{W^{\prime }(\text{A})-W^{\prime }(\text{B})}{\text{d}z+(W^{\prime }(\text{A})-W^{\prime }(\text{B}))}\right|=\frac{W^{\prime }(\text{A})-W^{\prime }(\text{B})}{\text{d}z+[W^{\prime }(\text{A})-W^{\prime }(\text{B})]}$$ (7)

When d*z*→0,

$$\phi (\text{A})=\underset{\text{d}z\to 0}{\lim}\frac{W^{\prime }(\text{A})-W^{\prime }(\text{B})}{\text{d}z+W^{\prime }(\text{A})-W^{\prime }(\text{B})}=\frac{\partial W^{\prime }(\text{A})/\partial z}{1+\partial W^{\prime }(\text{A})/\partial z}$$ (8)

Since point A is arbitrarily selected, the porosity of an arbitrary point in the gob can be expressed as

$$\phi (x,y,z)=\frac{\partial W^{\prime }(x,y,z)/\partial z}{1+\partial W^{\prime }(x,y,z)/\partial z}$$ (9)

When Eq (9) is inserted into the Blake—Kozeny equation, the spatial distribution function of gob permeability can be obtained as follows:

$$K(x,y,z)=\{\begin{array}{l} \frac{D_{\text{p}}^{2}}{150}\frac{\phi {\left(x,y,z\right)}^{3}}{{\left[1-\phi (x,y,z)\right]}^{2}}\\ \phi =0.0002522\exp\left(-\frac{z}{69.6}\right)y^{2}\left(1-{\left(\frac{x}{126}\right)}^{2}\right)\\ \times \exp\left[-0.002581\exp\left(-\frac{z}{69.6}\right)y^{2}\left(1-{\left(\frac{x}{126}\right)}^{2}\right)\right]\\ \left(0\leq y\leq 65\right)\\ \phi =0.0002522\exp\left(-\frac{z}{69.6}\right){\left(130-y\right)}^{2}\left(1-{\left(\frac{x}{126}\right)}^{2}\right)\\ \times \exp\left[-0.002581\exp\left(-\frac{z}{25.5}\right){\left(130-y\right)}^{2}\left(1-{\left(\frac{x}{126}\right)}^{2}\right)\right]\\ \left(65\leq y\leq 130\right) \end{array}$$ (10)

where *D*_p_ is the average particle diameter, which is taken as 0.2 m in this paper.

### 3.2 Spatial distribution of gob permeability

**Fig 9:**
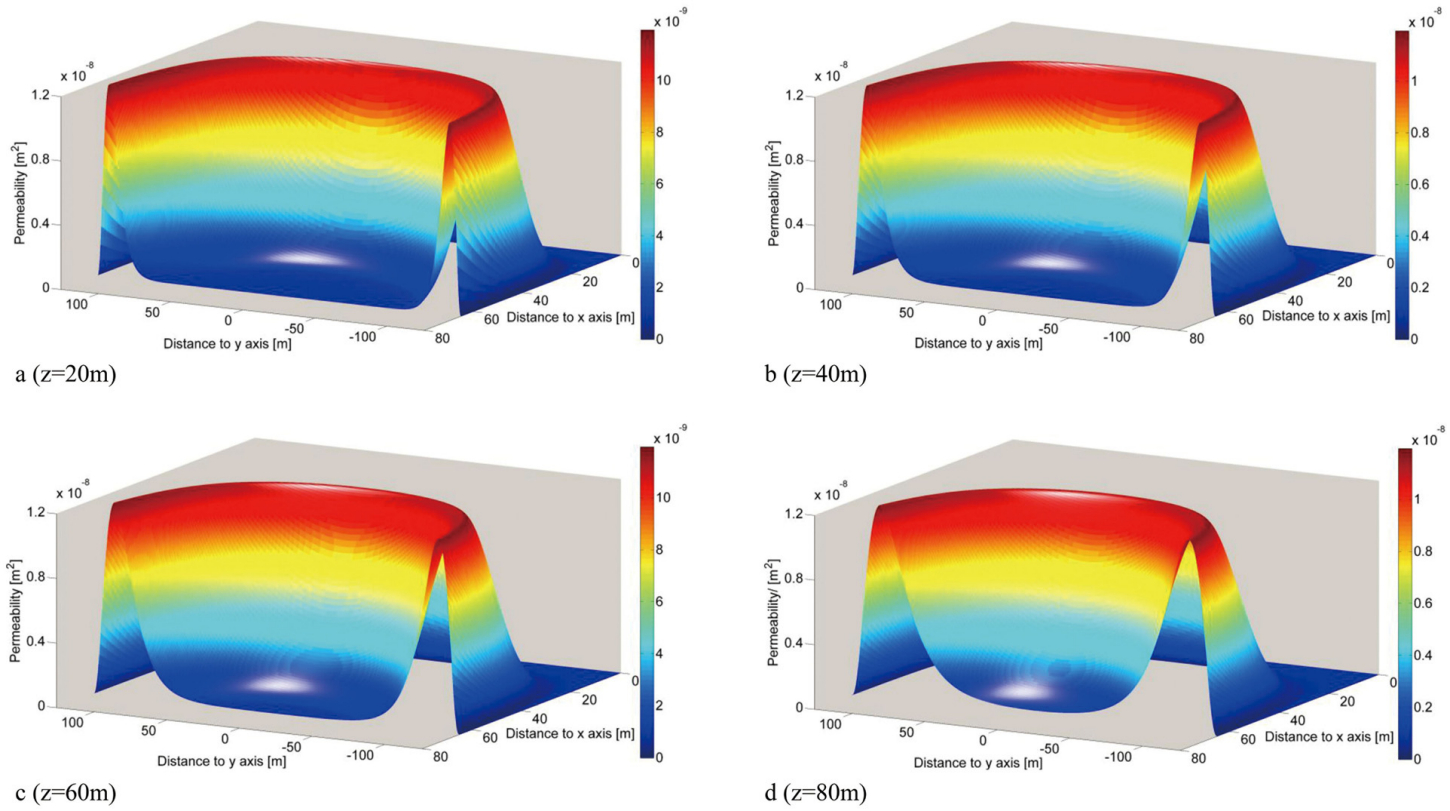
Permeability distribution in horizontal sections in the abandoned gob.

According to Eq (10), the distribution of gob permeability in the horizontal sections can be drawn for *z* = 20, 40, 60 and 80 m (Fig 9). For a more intuitive analysis of the change rule for gob permeability, only half of the model along the strike direction of the gob was considered in the analysis.

For the same horizontal section, Fig 9 shows that permeability is relatively higher near the area of the mining boundary than in the middle region of the gob mass. This is due to the supporting effect provided by the protective coal pillars that prevent the compaction of broken rock mass near the mining boundary. In contrast, the middle region of the gob mass is subjected to pressure from the overlying coal mass, and is well compacted, resulting in lower permeability. The zone of higher permeability near the area of the mining boundary appears as an ‘O’ shape in space, which is consistent with the O-ring theory of mining fissure [21]. It can be seen from the distribution diagram of permeability for different horizons that the range of the compacted zone is reduced from the bottom to the top, and the permeability within the compacted zone appears to be lower at the bottom and higher at the top.

## Numerical calculation

Large quantities of gob methane stored in the coal and rock are released during the coal seam mining process. Part of the gob methane is conveyed into the atmosphere through shafts, while some is drained directly by the gas drainage system. The remainder of the gob methane in the gob on cessation of underground coal mining operations is defined as abandoned gob methane. Abandoned gob methane could potentially be extracted from abandoned gob after the working face is closed. A numerical model using FLUENT for abandoned gob methane drainage through surface wells was established, and the derived spatial distribution function of gob permeability was imported into the numerical model. The control range of surface wells, gas flow patterns and distribution rules for static pressure in the gob under different well locations were determined using the calculated results from the numerical model.

### 4.1 Numerical calculation schemes

**Fig 10:**
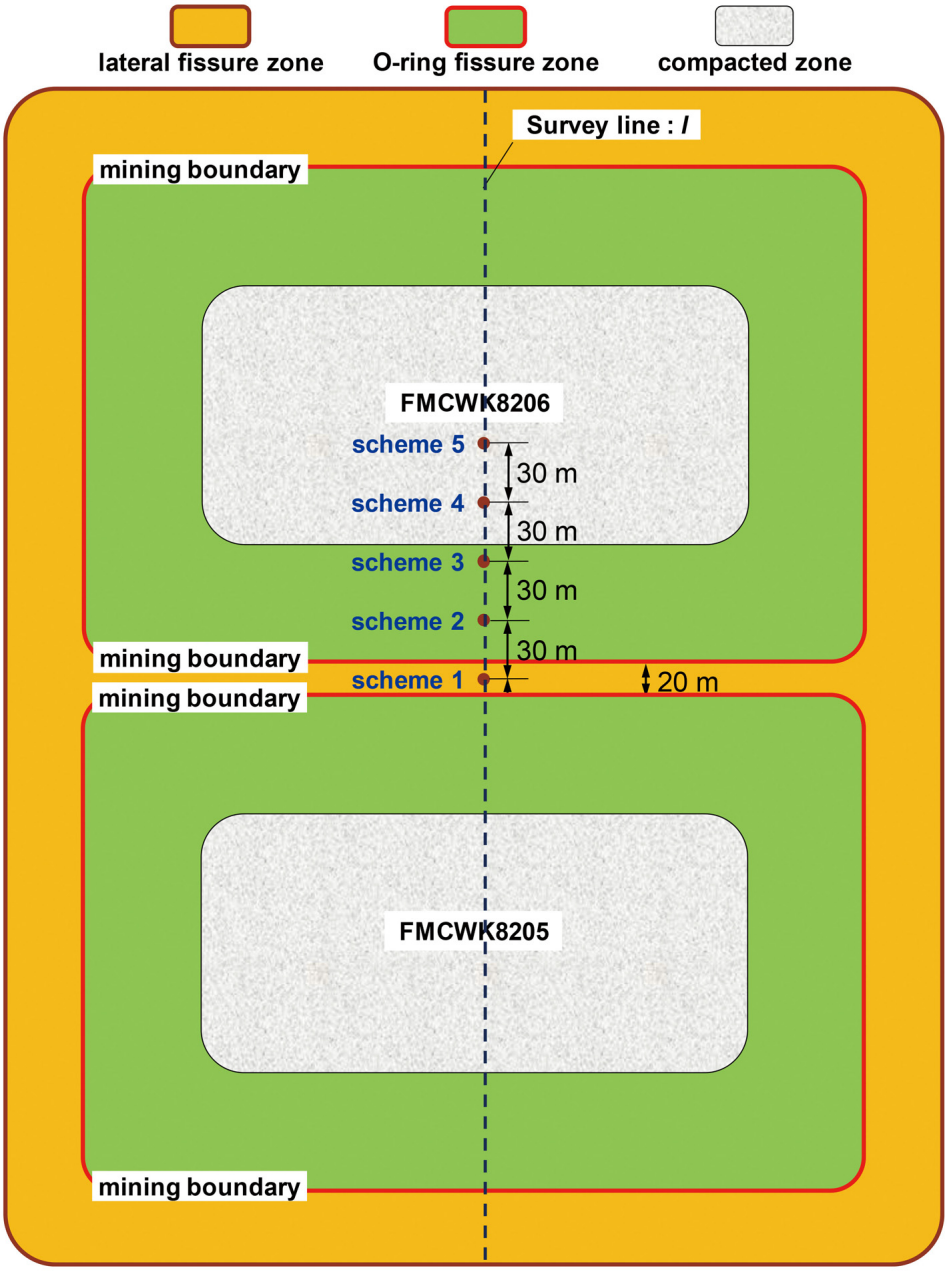
Regional division of the GCFZ.

According to the distribution of permeability in the horizontal sections of the gob, the GCFZ of FCMFK8206 was divided into a lateral fissure zone (outer regions of the mining boundary), an O-ring fissure zone (within 60 m of the mining boundary), and a compacted zone in the horizontal direction (Fig 10). Under the conditions of scheme 1, the surface well was located 10 m beyond the mining boundary of FMCWK8206. Based on scheme 1, the well locations of other schemes were moved 30 m inwards of the gob, in turn. Under the conditions of schemes 2 and 3, both surface wells were located within the O-ring fissure zone, where bed-separated fissures were developed with high permeability. Under the conditions of schemes 4 and 5, both surface wells were located within the compacted zone.

### 4.2 Fluid properties and boundary conditions set-up

**Fig 11:**
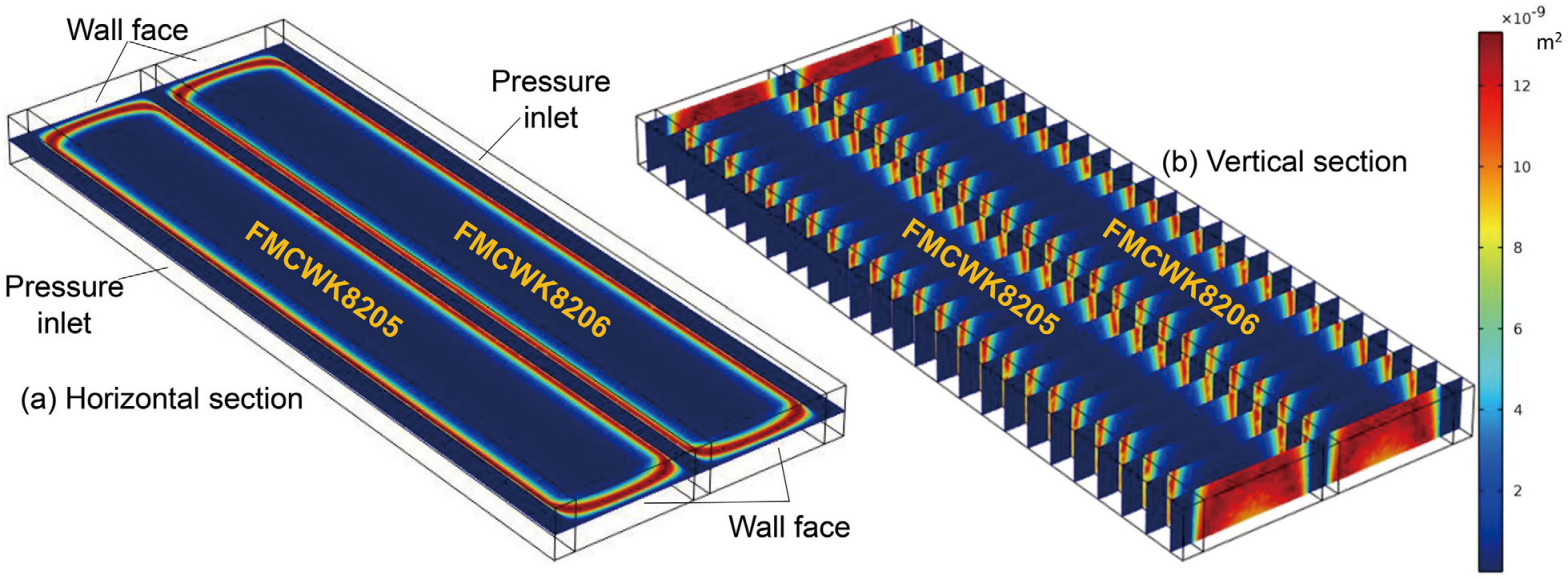
Permeability distribution of the model.

1. It was assumed that the methane mixed with air evenly in the abandoned gob in this model, and the density and dynamic viscosity of the air—methane mixture were taken as 0.648 kg/m^3^ and 11 *μ*Pa s, respectively.
2. The reference pressure was set as 101 325 Pa, and all results were relative pressures. The body of the surface well was set as the pressure outlet, and the suck pressure of the surface well was assigned a value of −10 000 Pa. The boundary faces of the coal pillar on both sides in the model were set as pressure inlets, with the pressure assigned as 0 Pa. All of the bottom plate, the top plate, the model boundary at the open-off cut, and the model boundary at the terminal line were set as wall faces (Fig 11).
3. The material in the abandoned gob was assumed to be a porous medium. The permeability in the abandoned gob was a continuous function, and a user-defined function for the spatial distribution of permeability in the GCFZ can be set according to Eq (10). The spatial distribution of gob permeability in this model is shown in Fig 11. From experimental results for gas seepage in coal with low permeability [22], the permeability in the lateral fissure zone in this model was taken as 1.6 × 10^–10^ m^2^.

### 4.3 Result and analysis

#### 4.3.1 Control range for the surface well at different well locations

In accordance with the modified equation for gas diffusion displacement and time [23], a gas flow velocity of 0.0003 m/s was set as the critical seepage velocity within the control range of the surface well in this study. The model was calculated using FLUENT.

The control range for the surface well at different well locations was obtained.

**Fig 12:**
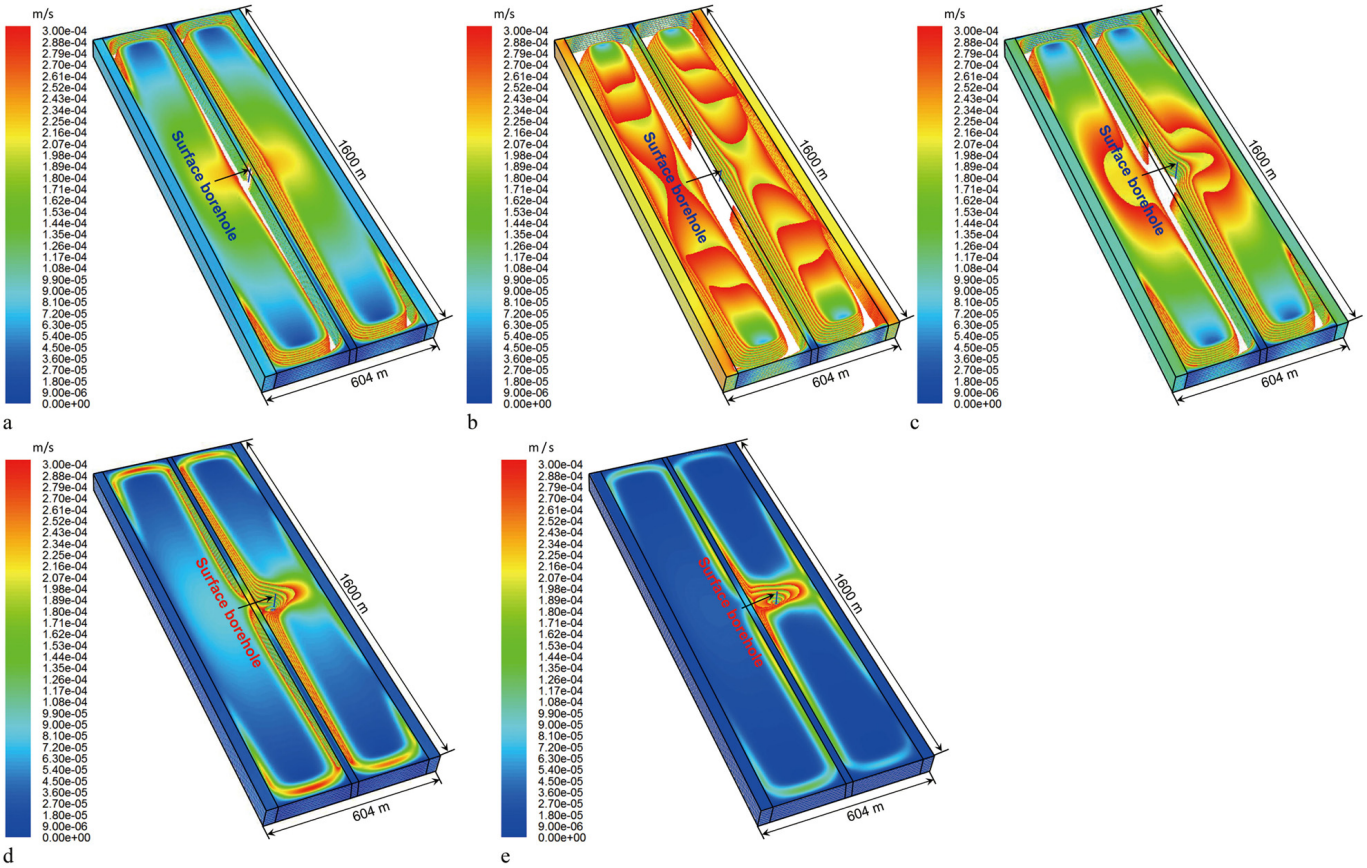
Control range for the surface well at different well locations.

1. Fig 12a showed that, under the conditions of scheme 1, the control range of the surface vertical well displayed a symmetric C-shaped distribution and encompassed the partial O-ring fissure zone on both sides of the middle coal pillar. The control effect on the gas in the compaction zones on both sides was weak.
2. Fig 12b illustrated that under the conditions of scheme 2, the control range of the surface vertical well was the largest, and the control range above the gob almost covered these two abandoned gobs.
3. It can be seen from Fig 12c, 12d, and 12e that, as the surface vertical well continuously goes deeper into the gob of FMCW K8206, its control range decreases constantly. At the same time, the control effect of the surface vertical well on the gob of FMCW K8205 weakened. In Fig 12e, the control range of the surface vertical well on the gob of FMCW K8205 disappeared.

#### 4.3.2 Flow patterns in the abandoned gob at different well locations

Fluid flow can be classified into two patterns: (1) laminar flow and (2) turbulent flow. When the gas is in a state of laminar flow in a porous medium, the fluid flows along the flow line parallel to the axis of the flow channel, without component velocity crossing the flow line. However, when the gas flows turbulently, the flow line of the fluid particle movement is disorderly. The laminar flow in the porous medium conforms to Darcy’s law, the gas flow rate is relatively small, and the gas flow rate is directly proportional to the pressure gradient. The turbulent flow in the porous medium does not conform to Darcy’s law, and the gas flow rate is relatively large and can be described with the nonlinear seepage theory. In gas drainage engineering practice, the gas flow state does not have significant influence on the gas drainage effect but it is crucial to the numerical calculation of velocity field, pressure field and gas concentration field in a gob. The Reynolds number is commonly used as a criterion to distinguish these two flow patterns. The Reynolds number can be expressed as

$$\text{Re}=\rho V\sqrt{K_{\text{p}}}/\mu$$ (11)

where *ρ* is the fluid density (kg/m^3^), *V* is the flow velocity (m/s), *K*_p_ is the permeability (m^2^) and *μ* is dynamic viscosity (Pa s).

Earlier studies [24] have shown that, for seepage flow with Re < 2300 through a porous medium, the flow is laminar. For this flow pattern, the seepage velocity and the pressure gradient are directly proportional, and the gas flow conforms to Darcy’s law. For seepage flow with Re > 2300, the flow gradually changes from laminar to turbulent. Darcy’s law is not applicable to this flow pattern.

**Fig 13:**
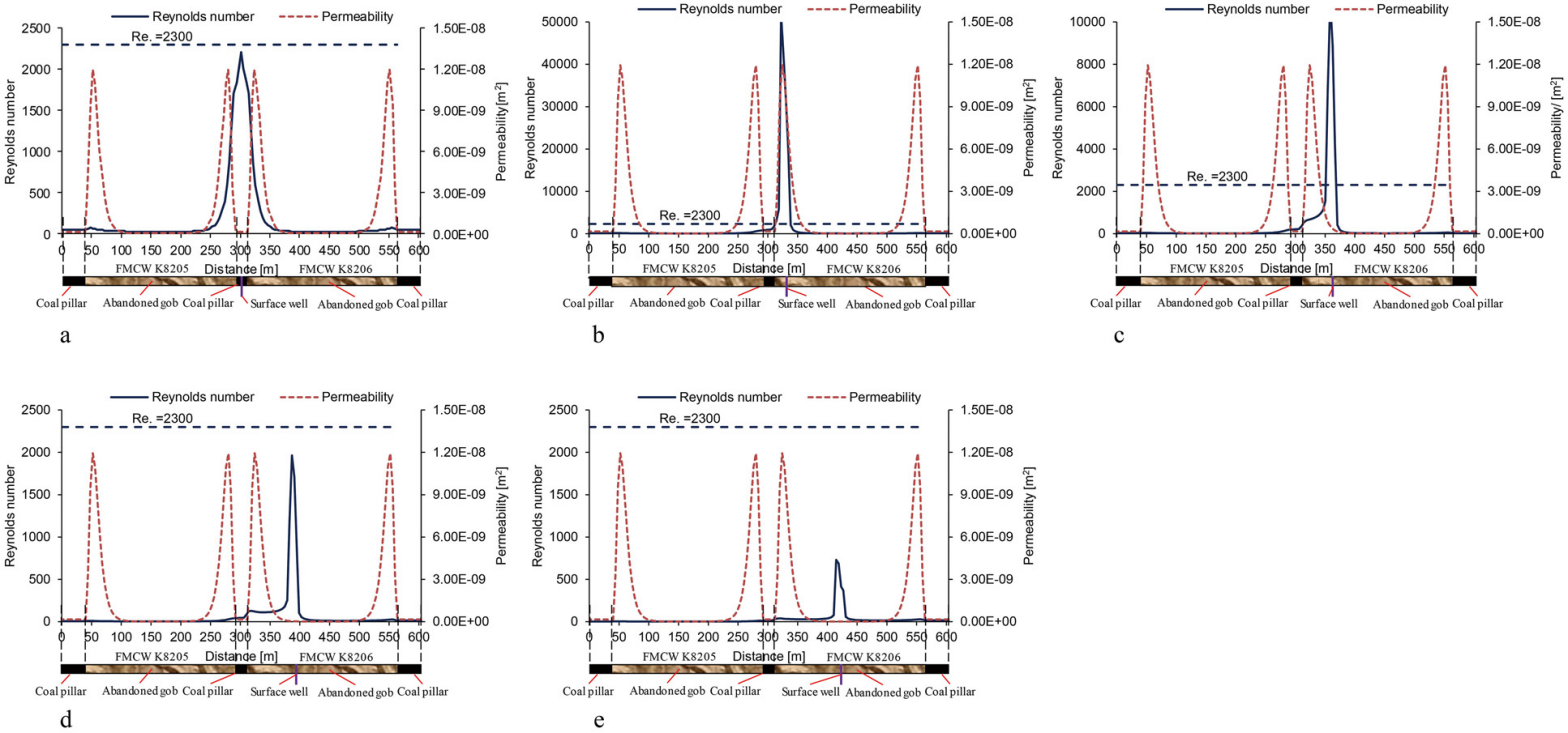
Distribution of the Reynolds number on the survey line at different well locations.

In order to investigate the flow patterns in the abandoned gob at different well locations, the Reynolds number on the geometric centre line *l* (Fig 10) of the model was obtained. The distribution of the Reynolds number is shown in Fig 13, in which Re = 2300 has been identified as the critical Reynolds number that distinguishes between laminar and turbulent flow patterns.

As shown in Fig 13, for the scheme 1 layout, when the surface well is in the lateral fissure zone, the Reynolds number for the flow near the surface well is higher compared with that further away from the surface well. However, the maximum Reynolds number on the survey line is still lower than 2300. Thus, when the surface well is in the lateral fissure zone, the flow pattern of the gas inside the abandoned gob is laminar.

For the scheme 2 and 3 layouts, when the surface well is in the O-ring fissure zone, the Reynolds number of the flow around the surface well is higher than 2300. Thus, when the surface well is located within the O-ring fissure zone, turbulent flow appears in the abandoned gob.

For the scheme 4 and 5 layouts, with the surface well deep in the compacted zone in the middle of the gob, the maximum Reynolds number on the centre line *l* decreased, and all the Reynolds numbers are lower than 2300. Thus, the flow pattern of the gas inside the abandoned gob is laminar.

The permeability distribution has significant influence on the shape of the Reynolds number curve. When the surface well is located in the compacted zone, the Reynolds number decreases sharply within the range of the compacted zone, whereas the decreasing trend becomes gentle within the range of the O-ring fissure zones, as shown in Fig 13c, 13d and 13e. The reason is that the gas flow rate decreases evidently in the low-permeability zone, leading to significant decrease in Reynolds number; however, the decrease amplitude of the gas flow rate is relatively small in the high-permeability zone, so the decrease amplitude of Reynolds number is relatively small.

#### 4.3.3 Distribution rules for static pressure in the gob at different well locations

**Fig 14:**
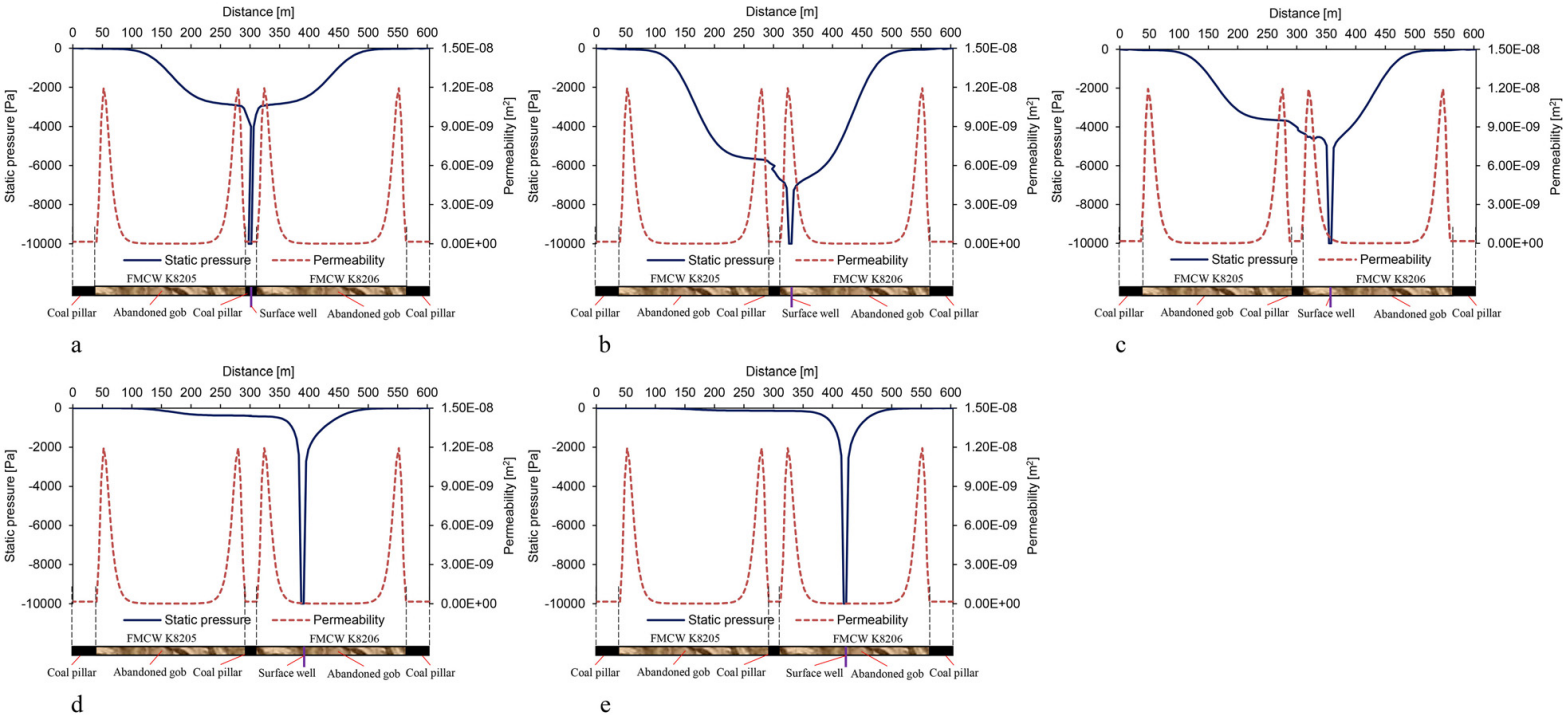
Distribution rules for static pressure in the gob at different well locations.

In order to investigate the distribution of static pressure inside the abandoned gob, the pressure data for the geometric centre line *l* of the model were obtained, as shown in Fig 14.

As shown in Fig 14a, when the surface well is in the lateral fissure zone, since the permeability in the lateral fissure zone is lower (1.6 × 10^–10^ m^2^), the negative pressure of extraction dropped quickly around the surface well. At the boundary of the gob, which is 10 m from the surface well, the negative pressure decreased from −10 to −3.39 kPa, and decreased by 66.1% within this 10 m. In this case, the pressure gradient inside the gob is lower, while the flow velocity of gas is slower, and the gas flow is laminar, which conforms to Darcy’s law.

As shown in Fig 14b and 14c, when the surface well is in the O-ring fissure zone, since the permeability in the O-ring fissure zone is higher, the attenuation of the negative pressure of extraction is therefore smaller. Under the scheme 2 and 3 conditions, the negative pressure 10 m from the surface well decreased from −10 to −6.89 kPa and −4.68 kPa, respectively, and decreased by 31.1% and 53.2%, respectively, in the 10 m range. In both cases, the static pressure gradient inside the gob is higher, the seepage velocity of gas flow in the O-ring fissure zone is faster, and turbulent flow appears in the area around the surface well, and the flow does not conform to Darcy’s law.

As shown in Fig 14d and 14e, with the surface well deep in the compacted zone in the middle of the gob, the negative pressure of extraction around the surface well dropped rapidly again. Under the scheme 4 and 5 conditions, the negative pressure 10 m from the surface well decreased from −10 to −1.83 kPa and −1.62 kPa, respectively, and decreased by 81.7% and 83.8%, respectively, within the 10 m range. In both cases, the pressure gradient inside the gob is lower, the seepage velocity of gas flow in the O-ring fissure zone is slower, and the gas flow is laminar, which conforms to Darcy’s law.

The permeability distribution also has great influence on the shape of the static pressure curve; when the surface well is located in the compacted zone, the static pressure attenuates relatively fast in the low-permeability zone but relatively slow in the high-permeability zone. Static pressure also exhibits similar change to that in Reynolds number (Fig 14c, 14d and 14e).

Based on the results from the model, when the surface well moves from the lateral fissure zone to the compacted zone in the middle of the gob, the permeability changes from low to high and then back to low again. The corresponding static pressure gradient in the gob changes from small to large and then back to small again, which causes the gas flow around the surface well to change from laminar to turbulent and back to laminar.

## Conclusions

A physical simulation was conducted using a three-dimensional simulation system for the gob in this study. The spatial distribution function for the vertical displacement of the overlying strata in the gob was obtained through analysis of the simulated result. On this basis, a spatial distribution function for gob permeability was derived, and imported into a numerical model. The control range of surface wells, flow patterns and distribution rules for static pressure in the abandoned gob at different well locations were determined by the calculation results from the numerical model.

The conclusions from this study are as follows:

1. The spatial distribution rule for gob permeability shows that the permeability near the area of the mining boundary is higher than in the middle region of a gob in the same horizontal section. Meanwhile, the distribution of higher permeability near the mining boundary area appears as an ‘O’ shape in space, which is consistent with the ‘O’ circle theory of mining-induced fracture.
2. By importing the spatial distribution function for gob permeability into the FLUENT software, the control range of a surface well at different well locations was obtained. When the surface well was located within 20 m of the mining boundary, the control range for the surface well was the largest. As the surface well continues to go deeper into the compacted zone, its control range decreases.
3. When the surface well is in the lateral fissure zone and the compacted zone, the gas flow inside the abandoned gob is laminar. When the surface well is in the O-ring fissure zone, turbulent flow appears around the surface well.
4. When the surface well moves from the lateral fissure zone to the compacted zone in the middle of the gob, the permeability changes from low to high and then back to low again. The corresponding static pressure gradient in the gob changes from small to large and then back to small again, which causes the gas flow around the surface well to change from laminar to turbulent and back to laminar.
